# Parathyroid Hormone Related Peptide (PTHrP) Revisited: A Comparison of Levels in Malignant and Non-malignant Conditions

**DOI:** 10.1007/s00223-026-01552-4

**Published:** 2026-06-10

**Authors:** Guoyu Ling, Rukayat Akande, Stewart G. Albert

**Affiliations:** Division of Endocrinology, Department of Internal Medicine, Saint Louis University School of Medicine, SLUCare Academic Pavilion, 1008 South Spring Street, St. Louis, MO 63110 USA

**Keywords:** PTHrP, Parathyroid hormone related peptide, Logistic regression analysis

## Abstract

Elevated parathyroid hormone-related peptide (PTHrP) levels are often presumed to indicate malignancy-associated hypercalcemia, yet PTHrP may be elevated in nonmalignant conditions. We compared PTHrP levels in patients with known malignancies with patients with elevations of PTHrP who had defined nonmalignant diagnoses to determine which levels of PTHrP were more indicative of malignancy. A retrospective review of electronic medical records in a large midwestern hospital system was conducted using search terms hypercalcemia with elevated PTHrP (> 2.3 pmol/L (men) and > 3.4 pmol/L (women). Binary logistic regression analyses assessed the positive predictive value (PPV, probability) of cancer. Among 236 patients with hypercalcemia and elevated PTHrP, 58 had known cancer. The others had defined nonmalignant diagnoses including primary hyperparathyroidism (n = 87), absorptive hypercalcemia (n = 28), and “other” causes (n = 63). PTHrP was highest in the cancer group (PTHrP = 36.9, 95% CI 19.8, 53.9 pmol/L, *p* < 0.001), however, overlap occurred. Binary logistic regression analyses showed a positive association of PTHrP levels (*p* < 0.001), while PTH levels were negatively associated (*p* < 0.001) with the presence of cancer. When analyzed together, PTH on PTHrP had independent effects (*p* < 0.001). At PTH ≥ 30 pg/mL, the PPV of cancer was < 5% when PTHrP < 7.0 pmol/L and < 10% at a PTHrP < 9.0 pmol/L. In the evaluation of hypercalcemia with elevated PTHrP, simultaneous levels of PTH measurements improve estimation of cancer probability. Mild PTHrP elevations with PTH > 30 pg/mL are more consistent with nonmalignant diagnoses, although higher PTHrP levels may still warrant evaluation for occult malignancy.

## Introduction

Primary hyperparathyroidism and hypercalcemia of malignancy are responsible for most causes of hypercalcemia [1–3]. Diagnostic protocols for hypercalcemia differ in their approaches. One approach begins with measurements of parathyroid hormone levels (PTH) alone [1] and if PTH is less than 15 pg/mL [3] or less than 30 pg/mL [2] to then obtain a test for parathyroid hormone related peptide (PTHrP) [2, 3]. Another approach suggests determining PTHrP only if there is suspected hypercalcemia of malignancy that had not been found after a diagnostic workup of mammography and computerized tomography [1]. It is stated that a PTHrP level greater than normal is highly suggestive of hypercalcemia of malignancy [2, 4].

PTHrP is a ubiquitous peptide found in diverse tissues, including breast, bone, cartilage, and inflammatory cells, and not only in cancers [1, 5, 6]. PTHrP binds to the PTHR1 receptor with many actions similar to PTH with regard to the effects of mobilizing bone, increasing renal cyclic AMP, and increasing renal calcium retention [6]. PTHrP however has a less potent effect on the generation of 1,25-dihydroxy vitamin D from 25-hydroxyvitamin D [1, 5–7].

In some incidences in the work up of hypercalcemia, clinicians may expediently order both PTH and PTHrP. In cases with elevated PTHrP without known malignancy, clinicians frequently follow with a diagnostic screening for unrecognized cancers [2, 4]. A principal publication asserted that elevated PTHrP was diagnostic of hypercalcemia of malignancy and elevated PTHrP was not found in normal subjects or in any other hypercalcemic condition [4]. In most cases of PTHrP associated hypercalcemia, the malignancy is advanced and clinically obvious [1]. However, when there is no previous knowledge of malignancy and PTHrP is elevated, there is the uncertainty of whether diagnostic screening for occult malignancy should be performed. Here we question whether PTHrP should be considered a marker for occult malignancy or whether there are diagnostic situations which would allow for the work up for unknown malignancy to be postponed while the diagnosis of hypercalcemia is re-evaluated.

## Methods

A retrospective review of the electronic health records (EPIC, Slicer/Dicer) of a large midwestern hospital system was performed with the search criteria of “hypercalcemia” (> 10.2 mg/dL) and “PTHrP above the normal range”. Inclusion criteria were men and women, age 21–80 years, both as inpatients and outpatients, tested during January 1, 2024, until December 31, 2024. Each chart was reviewed to confirm the presence of hypercalcemia and to validate the assigned diagnoses. Calcium values were not adjusted to albumin, as lower albumin concentration would confirm the presence of hypercalcemia. Ionized calcium measurements were unavailable, although these would also have verified elevated calcium levels.

Hypercalcemia diagnoses were established by the principal health care providers and categorized as malignancy-associated or non-malignancy associate. The diagnosis of non-cancer hypercalcemia was made by the principal provider either by confirming another cause of elevated calcium, or by a return of calcium to normal in follow up. Non-malignant comparators included primary hyperparathyroidism, absorptive hypercalcemia (elevated 25-hydroxy vitamin D, 1,25 dihydroxy vitamin D or excessive calcium intake) and “other” forms of hypercalcemia (examples are dehydration, immobilization, and tertiary hyperparathyroidism due to chronic renal disease). No new malignancies were detected in this cohort during the 1 year of the study.

Factors included in the analyses were: age, sex, body mass index (BMI), weight, serum levels of PTHrP, PTH, calcium, creatinine, estimated glomerular filtration rate (eGFR), magnesium, phosphorus, alkaline phosphatase, alanine aminotransferase (ALT), aspartate amino transferase (AST), albumin, 25-hydroxy vitamin D, and 1,25-dihydroxy vitamin D. All PTHrP determinations were performed at ARUP laboratories (500 Chipeta Way, Salt Lake City, UT, USA) by LC–MS/MS methodology which is unaffected by C-terminal or N-terminal metabolic byproducts of PTHrP [8]. The test was standardized in 236 healthy adults, age 20–67, using an epitope peptide (YLTQETNK) which has no homology with any other tryptic fragment of human proteins and is a specific surrogate marker for quantification of PTHrP [9]. The test sensitivity is 0.73 pmol/L [9]. The normal range of PTHrP is 0.0–2.3 pmol/L for men and 0.0–3.4 pmol/L for women [8].

All statistics were performed using SPSS (IBM version 29.0.0.0). Comparisons of parametric data were analyzed by ANOVA. Post hoc subgroup analyses were performed by Fisher’s Least Square method. Nonparametric data were analyzed by the Mann–Whitney U Test for comparison of two samples and Kruskal–Wallis Test with adjustment by the Bonferroni correction for multiple samples.

Binary logistic regression analyses were performed to assess for the relationship of PTHrP and cofactors on the positive predictive values (PPV) for cancer [10, 11]. Data is presented mean ± SD for parametric data and mean with 95% confidence interval (95% CI) for non-parametric data. Significance was defined as a *p* value < 0.05 by two-tailed testing.

## Results

There were 236 patients (120 men, 116 women, age 70.6 ± 12.8 SD years). Among them, 58 had known malignancy and were actively under therapy or palliation for cancer including cancers of: lung (n = 17), kidney (n = 5), head and neck (n = 5) breast [n = 4], bladder (n = 3), primary liver (n = 1), prostate (n = 1), pancreas (n = 1), and cervix (n = 1); multiple myeloma (n = 10), lymphoma (n = 4), melanoma (n = 1), and undefined cancers (N = 5). The non-malignant groups included Primary Hyperparathyroidism (n = 87); Absorptive hypercalcemia (n = 28); and “Other” (n = 63; dehydration n = 29, tertiary hyperparathyroidism n = 16, immobilization = 7, thiazide diuretics n = 6, adrenal insufficiency n = 2, lithium induced hyperparathyroidism n = 1, HIV n = 1, chronic hidradenitis inflammation n = 1), Table 1. No new cases of cancer were identified after the initial testing of the PTHrP levels.

**Table 1:** Clinical characteristics of patients with hypercalcemia and elevated PTHrP

	N	Cancer^a^	Hyperparathyroidism^b^	Absorptive^c^	Other^d^	P, ANOVA	Comparison
		58	87	28	63		
Age, years	Mean (SD)	69.4 (11.5)	73.1 (12.5)	72.5 (11.7)	67.4 (14.1)	0.37	
BMI, kg/m^2^	Mean (SD)	26.0 (7.1)	27.1 (6.4)	27.8 (6.1)	29.7 (6.9)	0.02	a < d
Creatinine, mg/dL	Mean (SD)	1.7 (1.2)	2.1 (1.8)	2.2 (1.2)	2.7 (2.5)	0.02	d > a
eGFR, mL/min/1.73 m^2^	Mean (SD)	57.4 (29.5)	50.2 (25.8)	42.7 (26.3)	46.6 (28.4)	0.025	a > c, d
Calcium, mg/dL	Mean (SD)	12.2 (1.6)	10.6 (1.0)	12.6 (2.4)	10.6 (1.6)	< 0.001	a > b, a > d
PTH, pg/mL	Mean (95% CI)	19.1 (13.6, 24.6)	170.1 (125.9, 214.4)	18.0 (11.9, 24.1)	103.5 (33.6, 173.4)	< 0.001	b > a, c; d > a, c
PTHrP, pmol/L	Mean (95% CI)	36.9 (19.8, 53.9)	4.6 (4.1, 5.0)	4.9 (3.8, 6.0)	4.63 (4.1, 5.2)	0.001	a > b, c,d
25,OH Vitamin D, ng/mL	Mean (SD)	32.8 (18.3)	39.5 (19.5)	64.2 (42.1)	38.8 (19.1)	< 0.001	c > a, b, d
1, 25 OH_2_ Vitamin D, pg/mL	Mean (SD)	37.1 (25.4)	44.6 (23.2)	87.2 (65.7)	29.7 (16.9)	< 0.001	c > a, b, d
Phosphorus, mg/dL	Mean (SD)	1.1 (0.2)	1.1 (01)	1.0 (0.2)	1.4 (0.2)	0.09	
Magnesium, mg/dL	Mean (SD)	2.0 (0.4)	2.3 (2.7)	2.0 (0.4)	1.9 (0.3)	0.68	
Albumin, g/dL	Mean (SD)	3.5 (4.5)	3.6 (0.7)	3.5 (0.7)	3.4 (0.9)	0.98	
Alkaline phosphatase, IU/L	Mean (SD)	153.2 (130.6)	92.2 (43.4)	92.4 (65.6)	124.4 (113.8)	< 0.001	a > b, c
alanine aminotransferase, IU/L	Mean (SD)	23.9 (28.0)	20.1 (18.1)	24.3 (16.6)	31.2 (27.8)	0.33	
Aspartate aminotransferase, IU/L	Mean (SD)	34.5 (38.0)	25.9 (18.9)	30.7 (3.9)	30.2 (16.5)	0.29	

Data show are mean ± SD for parametric data and median with 95% CI for non-parametric data. Comparisons significance was determined by ANOVA with significant difference among subgroups as per Methods

There were no differences among the groups in age, or serum levels of phosphorus, magnesium, albumin, alanine aminotransferase, or aspartate aminotransferase. As expected, the PTH levels were highest in the Primary Hyperparathyroidism group (PTH = 170.1, 95% CI 125.9, 214.4 pg/mL, *p* < 0.001). The Absorptive group had the highest serum 25 OH-Vitamin D (64.2 ± 42.1 ng/dL, *p* < 0.001) and serum 1,25 OH_2_-vitamin D (87.2 ± 65.7 pg/mL, *p* = 0.001). Alkaline phosphatase (153.2 ± 130.6 IU/L, *p* < 0.001) was highest in the Cancer group. Serum calcium levels were highest in the Cancer group (12.2 ± 1.6 mg/dL) and Absorptive hypercalcemia groups (12.6 ± 2.4 mg/dL) (*p* < 0.001).

**Fig. 1:**
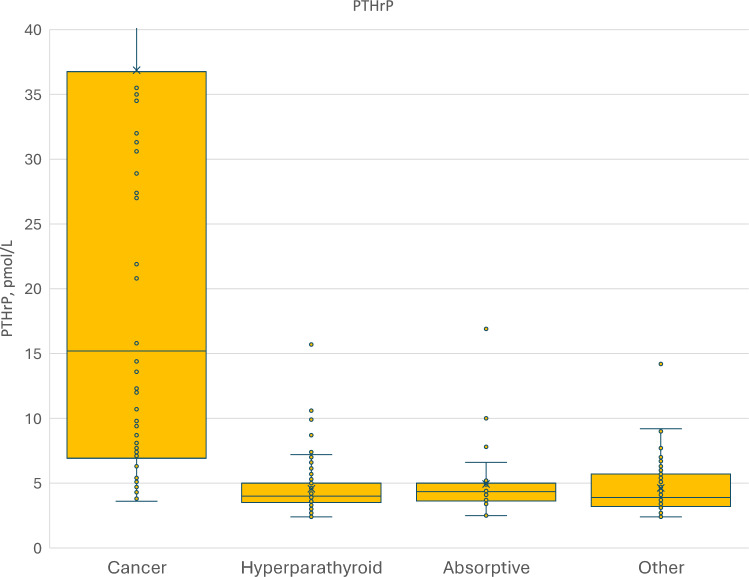
Comparison of subgroups of patients with hypercalcemia and elevated PTHRP levels as Box plots as median and interquartile ranges and whiskers at minimum and maximum values with outliers for cancer, primary hyperparathyroidism, absorptive hypercalcemia and “other”

**Fig. 2:**
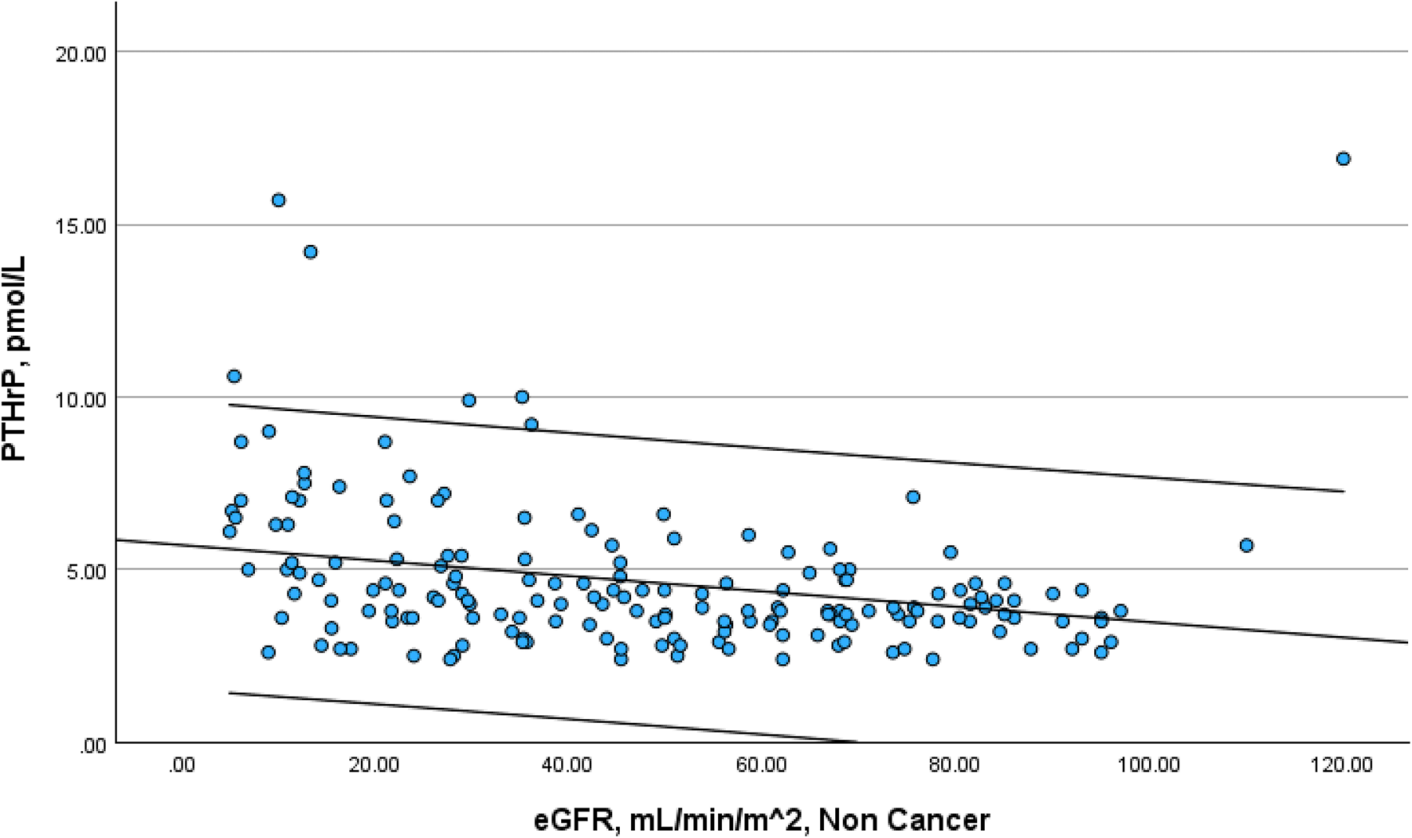
Regression analysis of PTHrP versus estimated glomerular filtration rate (eGFR) in patient without cancer. Shown are mean and 2 SD, where PTHrP = − 0.0229*(eGFR) + 0.571, r = − 0.275, *p* < 0.001)

The PTHrP was highest in the Cancer group (PTHrP = 36.9, 95% CI 19.8, 53.9 pmol/L) compared to the subgroups of Primary Hyperparathyroidism (PTHrP = 4.6, 95% CI 4.1, 5.0 pmol/L, *p* < 0.001), Absorptive hypercalcemia (PTHrP = 4.9, 95% CI 3.8, 6.0 pmol/L, *p* < 0.001) and Other hypercalcemia (PTHrP = 4.6, 95% CI 4.1, 5.2 pmol/L, *p* < 0.001). However, there were overlaps of PTHrP above the normal range in those with non-cancer associated hypercalcemia Fig. 1. In the non-cancer groups, all levels of PTHrP were elevated, as per the inclusion criteria, and there was a small but significant increase in PTHrP with decreases in eGFR, *p* < 0.001, Fig. 2. There were no correlations of PTHrP levels with age, sex, BMI, or race. There were 5 patients with PTHrP > 10 pmol/L in the non-cancer group, three had elevations of PTH (PTH = 355 pg/mL, 511 pg/dL, and 1582 pg/mL with concomitant renal insufficiency) and one had active inflammatory sarcoidosis (eGFR 56 mL/min/1.73 m^2^) and one had chronic hidradenitis inflammation (eGFR 120 mL/min/1.73 m^2^).

**Fig. 3:**
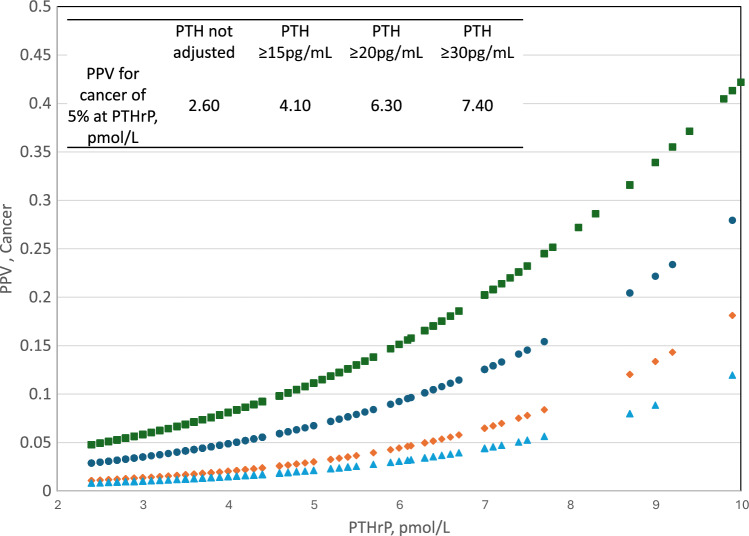
Binary logistic regression of probability (PPV) of cancer, versus PTHrP in pmol/L with categorical covariates: without considering simultaneous values of PTH (unadjusted for PTH, green squares) or in patients with simultaneous covariates of PTH ≥ 15 pg/mL (blue circles), PTH ≥ 20 pg/mL (red diamonds) and PTH ≥ 30 pg/mL (blue triangles). (The equation for logistic regression for PTHrP unadjusted for the level of PTH was PPV cancer = EXP^(Y)^/(EXP^(Y)^ + 1) where Y = (0.389*PTHrP) + (− 0.040*PTH) + (− 2.544), *p* < 0.001

To assess the comparison of PTHrP in those with and without malignancy with elevated PTHrP, binary logistic regression was performed. PTHrP levels were combined in all groups to determine the probability (positive predictive value, PPV) of cancer. There was a direct relationship of PTHrP with the positive predictive value of cancer (with the logistic equation—probability of cancer = Exp^(Y)^/(Exp^(Y)^ + 1, where Y = (0.352*PTHrP) − 3.84), *p* < 0.001), Fig. 3.

Statistical analyses typically use 5% as a reasonable threshold for decision-making. We selected a PPV of 5% (representing one in twenty chances of missing a cancer if one were present) as a practical threshold for determining whether to proceed with an immediate work up for malignancy or to defer evaluation. The level of PTHrP associated with PPV of cancer at a 5% level is shown without adjustment for PTH (PTHrP = 2.6 pmol/L). When concurrent PTH levels are included in the model, the PTHrP threshold for a 5% PPV for cancer increased to 4.10 pmol/L at PTH level of > 15 pg/mL, 6.1 pmol/L at PTH of > 20 pg/mL, and 7.4 pmol/L at PTH > 30 pg/mL, Fig. 3.

Stepwise logistic regression for other covariates along with PTHrP for the PPV of cancer did not show additive relationships over PTHrP alone for predicting cancer when considering age (*p* = 0.231), serum levels of calcium (*p* = 0.168), creatinine (*p* = 0.415), eGFR (*p* = 0.254), phosphorus (*p* = 0.524), ALT (*p* = 0.531), alkaline phosphatase (0.832), 25-hydroxyvitamin D (*p* = 0.187), or 1,25-dihydroxy vitamin D (*p* = 0.067).

## Discussion

In most cases of PTHrP derived hypercalcemia of malignancy, the underlying malignancy is advanced and clinically obvious [1]. In cases with elevated PTHrP without known malignancy, clinicians often must follow with diagnostic screening for occult cancers or other inflammatory conditions [2, 4]. In this study we evaluated patients who had elevated levels of PTHrP to see if elevated PTHrP is a marker for malignancy. Of the patients with elevated PTHrP above the normal range 25% had known malignancy, and no patient was newly diagnosed with an occult malignancy after obtaining the PTHrP laboratory results.

It is undetermined why patients with non-cancer mediated hypercalcemia had elevated PTHrP levels. PTHRP is a ubiquitous peptide found in inflammatory cells and many of these patients were hospitalized for intercurrent illnesses [1, 5, 6]. It is unlikely that the elevated PTHrP seen here was associated with metabolic fragments as had been seen in previous N-terminal or C-terminal immunoassays [12, 13]. All testing was performed by the same laboratory by LC–MS/MS assay which is unaffected by these metabolic fragment metabolites [8]. There was a small but significant increase in PTHRP with declining eGFR. We cannot say whether there is a uniform increase in PTHrP in all patients with diminished eGFR since we did not obtain PTHrP levels prospectively over all ranges of eGFR.

There are limitations to this study. Statistical tests typically use 5% as a reasonable threshold for decision making. We chose a PPV of < 5% for having cancer as a practical threshold for deferring workup and for reconsidering alternative causes of hypercalcemia. This PPV of 5% should not be considered as an absolute threshold and would be subject to the likelihood of other causes of hypercalcemia, comorbidities, age and shared decision of patient and provider. It is possible that there were concomitant elevations of PTHrP and calcitriol in patients with cancer, although that has been reported as exceeding rare, much less than the approximate 10% of patients with absorptive hypercalcemia found here [14–16]. Chukir et al. [14] were able to evaluate 101 patients during 9 years follow up at a major cancer center at Memorial Sloan Kettering Cancer Center out of their total patient population with known cancer (not stated). Sheehan et al. [16] performed a retrospective review of 167,551 patients with cancer and were able to identify 153 cases of concomitant elevations of PTHrP and calcitriol. Kometas and Maalouf [15] concluded in their review of humoral hypercalcemia of malignancy that the impact of PTHrP on calcitriol synthesis remains controversial.

It is possible that some of the patients could have demonstrated occult cancers in follow up as the study was performed over a 1-year timespan. However, patients had identification of other causes for hypercalcemia or resolution of hypercalcemia in follow up which would not be typical for hypercalcemia of malignancy.

In conclusion, these calculations for positive predicted values were dependent upon the prevalence of hypercalcemia of malignancy in our study populations, which may be different from other populations. Since the inclusion criteria was for patients with elevated PTHrP, we cannot say whether there is a uniform increase in PTHrP in all patients with diminished eGFR since we did not obtain PTHrP levels prospectively over all ranges of eGFR. It is clear from our analysis that there is a reciprocal relationship between PTHrP and PTH in association with the probability of hypercalcemia of malignancy. The concurrent determinations of PTH and PTHrP may provide estimations for the probability of finding an occult cancer and would allow for the postponement of and re-evaluation for other causes of hypercalcemia.
